# Acute right brachial artery embolism after left upper division segmentectomy with suspected pulmonary vein stump thrombus

**DOI:** 10.1093/jscr/rjag811

**Published:** 2026-09-16

**Authors:** Sotaro Takemura, Taiki Sato, Kazuki Sato, Hirofumi Uehara, Masahiro Miyajima

**Affiliations:** Department of Thoracic Surgery, Hakodate Goryoukaku Hospital, 38-3 Goryokakucho, Hakodate, Hokkaido 040-8611, Japan; Department of Thoracic Surgery, Hakodate Goryoukaku Hospital, 38-3 Goryokakucho, Hakodate, Hokkaido 040-8611, Japan; Department of Thoracic Surgery, Hakodate Goryoukaku Hospital, 38-3 Goryokakucho, Hakodate, Hokkaido 040-8611, Japan; Department of Thoracic Surgery, Hakodate Goryoukaku Hospital, 38-3 Goryokakucho, Hakodate, Hokkaido 040-8611, Japan; Department of Thoracic Surgery, Sapporo Medical University School of Medicine, South 1 West 16, Chuo-ku, Sapporo, Hokkaido 060-8543, Japan

**Keywords:** acute limb ischemia, brachial artery embolism, lung segmentectomy, postoperative embolism, pulmonary vein stump, anticoagulation

## Abstract

Systemic arterial embolism is an uncommon but potentially serious complication after lung resection. A 63-year-old woman underwent thoracoscopic left upper division segmentectomy for lung adenocarcinoma. On postoperative day (POD) 2, she developed sudden pain, numbness, and weakness of the right upper limb. Bedside ultrasonography showed right brachial artery occlusion, and contrast-enhanced computed tomography (CT) confirmed a distal brachial artery filling defect. A small filling defect was also observed at the pulmonary vein stump, but the embolic source could not be established. Emergency thrombectomy using a Fogarty catheter restored arterial perfusion ~4 h after symptom onset, followed by anticoagulation. Contrast-enhanced CT on POD 28 showed resolution of the pulmonary vein stump filling defect, and no recurrent embolism or bleeding occurred during 3 months of follow-up. This case highlights that sudden upper-limb symptoms after left upper division segmentectomy should prompt immediate vascular assessment, timely revascularization, and evaluation of possible embolic mechanisms.

## Introduction

Systemic arterial embolism is an uncommon but potentially serious complication after lung resection. Although postoperative embolic events are often discussed in relation to cerebral infarction after left upper lobectomy, embolism presenting as acute limb ischemia after segmentectomy is less commonly recognized. In particular, after left upper division segmentectomy, several mechanisms may need to be considered, including perioperative atrial fibrillation (AF), postoperative hypercoagulability, malignancy-associated thrombosis, coronavirus disease 2019 (COVID-19)-associated coagulopathy and, in selected cases, a pulmonary vein stump thrombus (PVST) as a possible embolic source. We report a case of acute right brachial artery embolism early after left upper division segmentectomy, emphasizing the need for prompt vascular assessment while considering multiple possible embolic mechanisms.

## Case presentation

**Figure 1:**
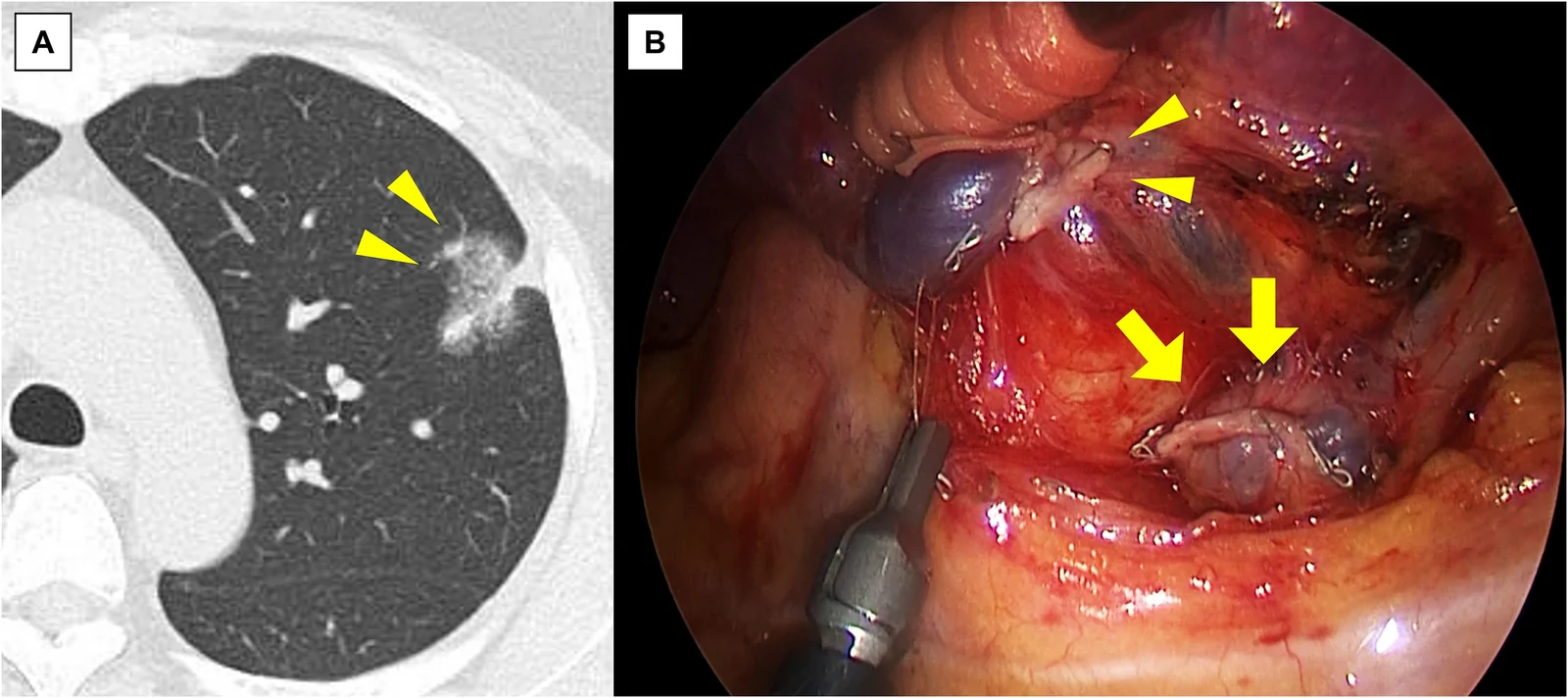
Preoperative CT and intraoperative division of the left V1 + 2 and V3 pulmonary veins. (A) Preoperative chest CT showing a 28-mm part-solid nodule with an 8-mm solid component in the left upper lobe, indicated by arrowheads. (B) The V1 + 2 and V3 pulmonary veins were divided using an endoscopic stapler. Arrowheads indicate the venous stump of the resected specimen. The arrow indicates the cardiac-side remnant pulmonary venous stump of the common trunk formed by the left V1 + 2 and V3.

**Figure 2:**
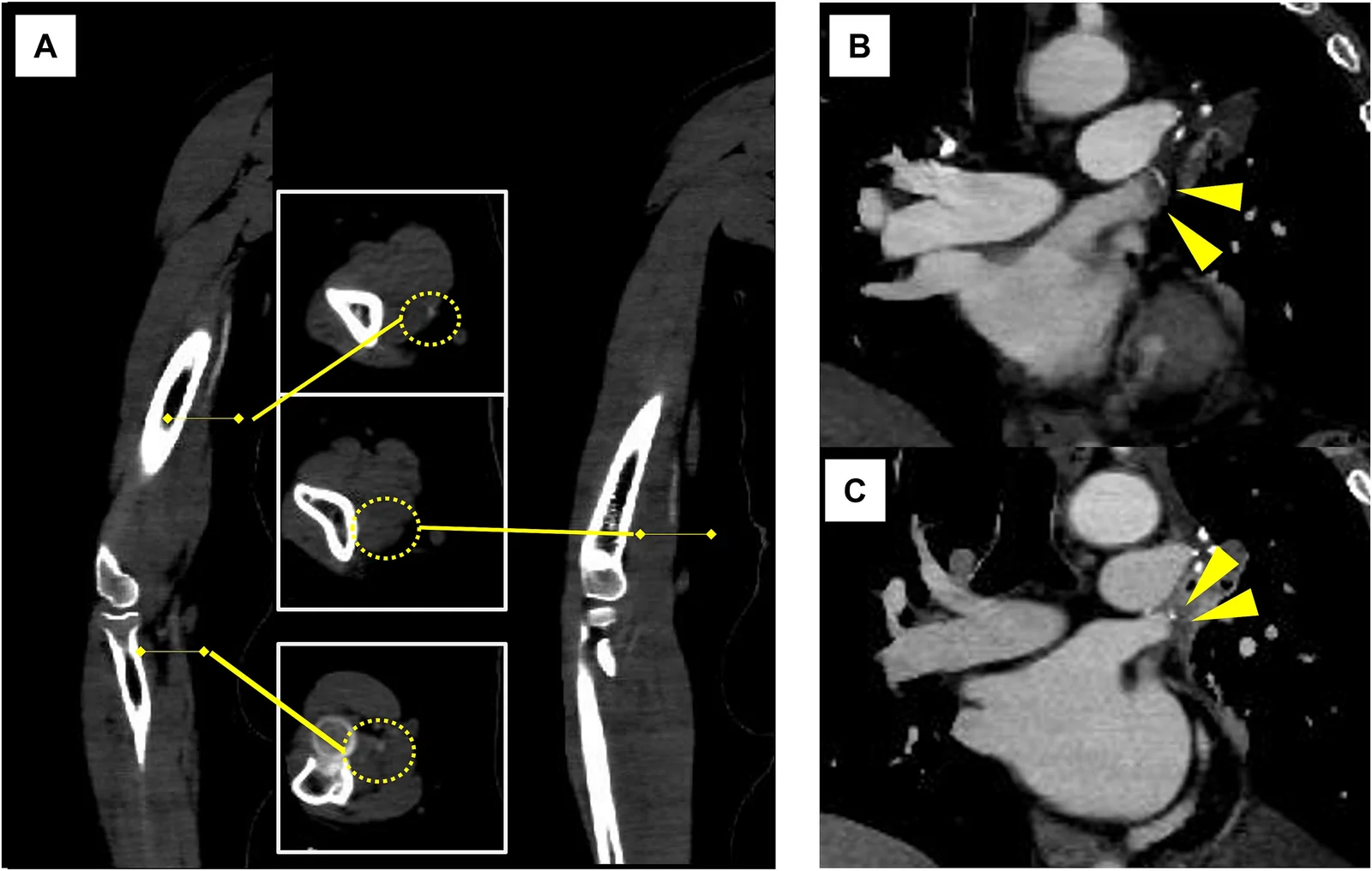
Contrast-enhanced CT findings at the onset of right brachial artery embolism and at follow-up. (A) Contrast-enhanced CT shows occlusion of the right brachial artery near the distal humerus, with collateral opacification of the radial and ulnar arteries distal to the occlusion. (B) Contrast-enhanced CT performed immediately after the onset of right brachial artery embolism on postoperative day 2 shows a small filling defect near the left upper-division pulmonary vein stump, suggestive of pulmonary vein stump thrombosis after left upper division segmentectomy. (C) Contrast-enhanced CT on postoperative day 28 shows resolution of the filling defect, with no residual thrombus in the left upper-division pulmonary vein stump.

A 63-year-old woman was referred for evaluation of a pulmonary nodule in the left upper lobe. She had no history of thromboembolism or AF. Her preoperative D-dimer level was <0.5 μg/mL. Chest computed tomography (CT) showed a left upper-lobe nodule measuring 28 mm in total diameter with an 8-mm solid component (Fig. 1A). Transbronchial biopsy revealed adenocarcinoma, and the clinical stage was cT1aN0M0, stage IA1. The patient underwent video-assisted thoracoscopic left upper division segmentectomy with lymph node sampling of stations #5 and #6. The left upper-division pulmonary veins, V1 + 2 and V3, were divided using an endoscopic stapler (Fig. 1B). The remaining pulmonary arterial branches, bronchus, and intersegmental planes were divided using staplers. Pathological examination revealed invasive adenocarcinoma, pT1cN0M0, stage IA3. Continuous electrocardiographic (ECG) monitoring was maintained in the intensive care unit until postoperative day (POD) 1 and showed no arrhythmia. On POD 2, after transfer to the general ward, the patient developed sudden pain, numbness, and motor weakness of the right upper limb. She also tested positive for COVID-19 on the same day, but the infection was clinically mild, without requiring supplemental oxygen or causing postoperative respiratory deterioration. Bedside ultrasonography showed thrombotic occlusion of the right brachial artery with absent distal flow. Contrast-enhanced CT demonstrated occlusion of the distal brachial artery, with collateral opacification of the radial and ulnar arteries (Fig. 2A). The same examination showed a small filling defect in the pulmonary vein stump, suggestive of a small thrombus (Fig. 2B). A repeat ECG showed sinus rhythm without AF. The D-dimer level at symptom onset was 1.3 μg/mL. Emergency thrombectomy was performed under local anesthesia through a right elbow incision. A fresh, dark-red thrombus measuring ~10 mm in length was removed from the proximal brachial artery using a 3-Fr Fogarty catheter, and arterial perfusion was restored ~4 h after symptom onset. Intravenous heparin was started after thrombectomy and switched to oral edoxaban on POD 3. Histopathological examination confirmed a fresh thrombus. Follow-up Doppler ultrasonography performed on 3 consecutive days confirmed patency of the brachial, radial, and ulnar arteries. The patient was discharged on POD 6. Contrast-enhanced CT on POD 28 showed resolution of the filling defect at the pulmonary vein stump (Fig. 2C). Because no recurrent thromboembolic event or residual thrombus was observed, edoxaban was discontinued on the same day. Three months after surgery, the patient had no symptoms suggestive of recurrent embolism or bleeding complications.

## Discussion

This case highlights two clinically important points. First, PVST may represent a rare but relevant thromboembolic complication after left upper division segmentectomy. PVST has been reported mainly after left upper lobectomy, but previous studies have also shown that thrombus can occur in the pulmonary vein stump after left upper division segmentectomy [1–3]. In the present case, contrast-enhanced CT at the onset of acute limb ischemia showed a small filling defect in the pulmonary vein stump, and contrast-enhanced CT on POD 28 confirmed its resolution. Although this finding does not prove a direct causal relationship with the brachial artery embolism, it is compatible with PVST as a possible embolic source after left upper division segmentectomy. Second, this case emphasizes the importance of rapid recognition and treatment of postoperative arterial embolism. The patient developed sudden pain, numbness, and motor weakness of the right upper limb on POD 2. These symptoms could have been misinterpreted as postoperative neuropathy due to intraoperative positioning. However, bedside ultrasonography immediately demonstrated brachial artery occlusion, leading to prompt contrast-enhanced computed tomography evaluation and emergency thrombectomy. Arterial backflow was restored ~4 h after symptom onset, and no recurrent embolism or bleeding complication occurred during follow-up. This clinical course illustrates that early vascular assessment is essential when sudden limb symptoms occur after thoracic surgery [4, 5]. The embolic mechanism should nevertheless be interpreted with caution. PVST was suspected radiologically and later disappeared, but the actual embolic source could not be proven. Perioperative AF is another important cause of systemic arterial embolism after thoracic surgery [6]. Although continuous ECG monitoring until POD 1 and a repeat ECG at symptom onset showed no AF, transient paroxysmal AF after transfer to the general ward cannot be completely excluded. Postoperative hypercoagulability, malignancy-associated thrombosis, and COVID-19-associated coagulopathy may also have contributed [7]. The patient tested positive for COVID-19 on the day of symptom onset, although the infection was clinically mild without oxygen requirement or respiratory deterioration. The limitations of this report include its single-case design, the absence of pathological confirmation of the suspected PVST, discontinuation of continuous ECG monitoring before symptom onset and the lack of detailed thrombophilia testing. Therefore, this case should not be interpreted as definitive evidence that PVST caused the brachial artery embolism. Rather, it provides a practical message for postoperative management: after left upper division segmentectomy, PVST should be included among possible causes of systemic embolism, and sudden upper-limb symptoms should prompt immediate vascular evaluation and timely revascularization.
